# The Breaking Point and Post-Traumatic Growth in Breast Cancer Survivors

**DOI:** 10.3390/cancers15184441

**Published:** 2023-09-06

**Authors:** Antonio Franco, Stefano Magno

**Affiliations:** Breast Unit, Department of Women, Children and Public Health Sciences, Fondazione Policlinico Universitario “A. Gemelli” IRCCS, 00168 Roma, Italy; antonio.franco@guest.policlinicogemelli.it

Advancements in breast cancer survival rates make the issues of quality of life and psycho-physical wellbeing in survivors central goals of comprehensive care.

Anticancer treatments, along with outstanding results on prognosis [1], also carry burdensome toxicities for every aspect (physical, emotional, social, or financial) of a woman’s life, as well as side effects and sequelae, even in the long term [2,3].

Unfortunately, the long-lasting consequences of a cancer journey on body image and weight [4,5], physical function [6], sexual health [7,8], psychological balance [9,10], and spiritual issues are hugely underdiagnosed and these patients’ needs are rarely met.

The health questions that remain unanswered during the survivorship trajectory hamper complete recovery after cancer experience and, in some cases, represent a threat to the survivor’s chances of cure. In particular, the increased rates of being overweight and sedentary habits in breast cancer survivors have been shown to negatively affect not only their quality of life, but also their disease-free survival, secondary cancers, and cardiovascular disease rates [11,12,13].

Despite this, supportive therapies and lifestyle remodulation programs are almost never proposed as a standard of care post-diagnosis, and the quality of life of cancer survivors has long been neglected in daily practice and clinical trials, until recent times [14].

The present Special Issue focuses on these topics, trying to shed light on some of the evidence-based resources in the field. 

Furthermore, there is still an insufficient and patchy use of patient-reported outcome (PRO) measures and the patient’s perspective is seldom considered in defining the primary outcomes in clinical studies, with a consequent underestimation of the toxicities and goals that matter to cancer patients [15,16].

When diagnosed with cancer, every woman has to face a breaking point, from which there will be a life “before” and “after” that moment. Along the cancer journey, underrating the negative impact that lifesaving anticancer treatments will unavoidably have on her body–mind balance, self-efficacy, and overall wellbeing will reduce the patient’s compliance to treatments and could, in some cases, convince her to abandon evidence-based therapies for unproven and dangerous alternative proposals.

An integrative holistic approach to every breast cancer patient, since diagnosis, should include an assessment of her lifestyles (nutrition, physical activity, sleep), psychological resources, and needs in order to improve her coping, enhance adherence to the treatment protocols, and reduce their side effects, even through non-pharmacological treatments [17,18].

This approach must be safe, rational, and evidence-based, tailored to the patient’s needs and preferences, according to the healthcare professional experience, and possibly provided in the same center where the oncological treatments are given.

In the first phases, prehabilitation to the active treatments, such as surgery and chemotherapy, may be needed; even small modifications in dietary and physical activity habits, smoking and alcohol cessation or reduction, relaxation strategies, sleep hygiene, and medical optimization just before or during these therapies could reduce perioperative complications and improve physical and psycho-social outcomes [19]. In the long term, the best chances for rehabilitation and supportive care must be provided in order to prevent and alleviate symptoms and sequelae along the pathway of care.

By helping the patient to remodulate her habits, when needed, during and beyond the cancer trajectory, she feels involved in a more proactive follow up, which becomes not only a period of surveillance for disease relapses, but also a window of opportunity for oncologists, psycho-oncologists, and other professionals to prescribe, motivate, and monitor positive behavioral changes in a comprehensive risk-reducing strategy.

By using arts for therapeutic purposes, such as music, dance, writing, and figurative arts, under the supervision of trained professionals, the individual experience of cancer can be told, manipulated, and transformed into something that could help to find a sense to one’s suffering.

Eventually, by recognizing and sustaining individual resources, an integrative model could help survivors to find a new balance for the rest of their lives (no matter how long) and enhance their post-traumatic growth beyond the disease [20].
